# Single-Molecule Imaging of Integral Membrane Protein Dynamics and Function

**DOI:** 10.1146/annurev-biophys-070323-024308

**Published:** 2024-07

**Authors:** Arnab Modak, Zeliha Kilic, Kanokporn Chattrakun, Daniel S. Terry, Ravi C. Kalathur, Scott C. Blanchard

**Affiliations:** 1 Department of Structural Biology, St. Jude Children’s Research Hospital, Memphis, Tennessee, USA; 2 Department of Chemical Biology & Therapeutics, St. Jude Children’s Research Hospital, Memphis, Tennessee, USA

**Keywords:** single-molecule imaging, integral membrane proteins, fluorescence resonance energy transfer, genetic code expansion, metastable energy landscape, intrinsically dynamic systems

## Abstract

Integral membrane proteins (IMPs) play central roles in cellular physiology and represent the majority of known drug targets. Single-molecule fluorescence and fluorescence resonance energy transfer (FRET) methods have recently emerged as valuable tools for investigating structure–function relationships in IMPs. This review focuses on the practical foundations required for examining polytopic IMP function using single-molecule FRET (smFRET) and provides an overview of the technical and conceptual frameworks emerging from this area of investigation. In this context, we highlight the utility of smFRET methods to reveal transient conformational states critical to IMP function and the use of smFRET data to guide structural and drug mechanism-of-action investigations. We also identify frontiers where progress is likely to be paramount to advancing the field.

## INTRODUCTION

Integral membrane proteins (IMPs) represent approximately 20–30% of the expressed proteomes in all kingdoms of life (7). IMPs are synthesized by ribosomes and typically fold into their three-dimensional structures within endoplasmic reticulum lipid bilayers through cotranslational and post-translational processes (69). IMPs are subsequently transported to their destination membranes (either plasma or organellar) by vesicular (37, 175) and nonvesicular pathways (68). During transit, post-translational modifications may be added (94). Within chemically diverse and compositionally distinct membrane barriers (115, 162), IMPs govern myriad physiological processes ranging from cell signaling, solute and ion transport, metabolism, and membrane remodeling processes to changes in membrane leaflet composition and orientation, including endo- and exocytosis. The principal IMP superfamilies are broadly divided into solute carrier proteins (SLCs); ion channels (ICs); ATP-binding cassette (ABC) transporters; receptor tyrosine kinases; G protein–coupled receptors (GPCRs); and integral membrane enzymes such as the F-type, P-type, and V-type ATPases (140). Congruent with their essential cellular roles and residence within periphery barrier membranes, IMPs represent approximately 60% of known drug targets (144).

The functions of IMPs are fundamentally linked to their dynamic properties, such as diffusion within their two-dimensional bilayer environments, time-dependent changes in conformation and composition, or both. Covering all aspects of IMP dynamics is beyond the scope of any single review. In this review, we narrow the discussion to experimental observations of the dynamic properties of polytopic IMPs, the largest subset of IMPs, defined as those exhibiting two or more lipid bilayer transmembrane-spanning segments (6). Polytopic IMPs adopt distinct structural topologies evolved to mediate mechanistically diverse processing and transfer of information from one physiologically distinct compartment to the next. Their functions contribute to growth, development, and survival (186) by playing roles in essential life processes, including the regulated transport of ions, small molecules, and larger cargo between the external environment and the inside of the cell, as well as between internal organelles and the cytoplasmic milieu.

We specifically focus on the motivation, practical implementation, and experimental interrogation of polytopic IMP functions using single-molecule fluorescence resonance energy transfer (smFRET) methods. Using smFRET, experimentalists have revealed that basic and regulated IMP functions are governed by the principles of metastability. The metastability concept was first introduced by the protein folding community to explain alternative protein folding pathways and was later extended to and further developed for fully folded systems (72, 75, 105, 135, 137, 143, 173). Contemporary metastability frameworks hold that the functions of IMPs (polytopic or otherwise), like those of other biomolecules, are intrinsically linked to their evolved capacity to spontaneously transition among multiple distinct native state folds. Energetic differences between local minima on this metastable energy landscape are small enough, and thus transitions between states are rapid enough, to be strongly influenced by mutations and modifications, as well as exogenous variables such as temperature, solvent, and ligand binding.

**Figure 1:**
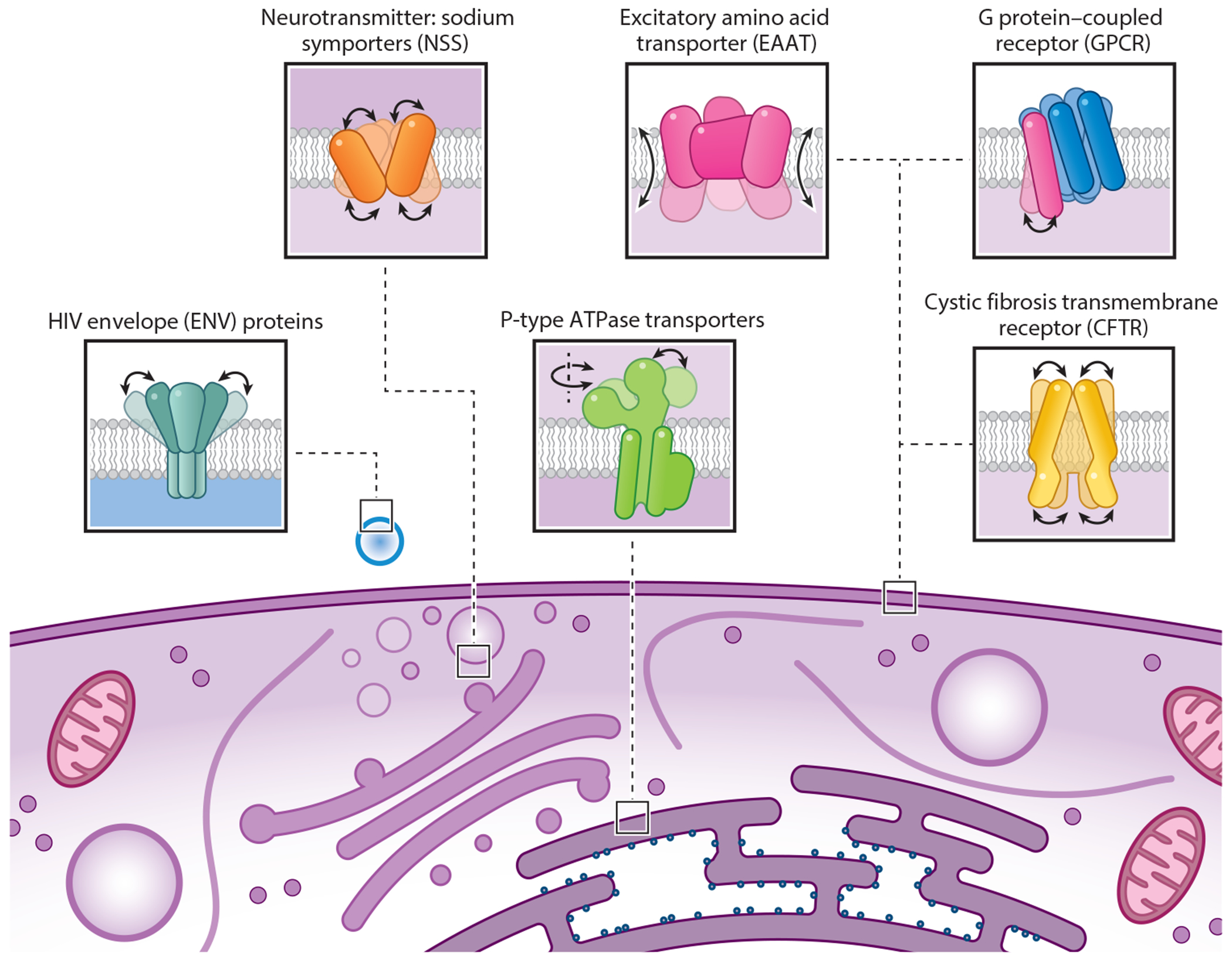
Polytopic integral membrane proteins (IMPs) investigated by single-molecule fluorescence resonance energy transfer (smFRET) in their physiological settings. Shown are schematic representations of different types of IMPs, residing in the plasma or the organellar or viral membrane, that were investigated by smFRET to understand the link between their dynamics and function.

In close collaboration with experts in specific polytopic IMPs, we have examined structure–function relationships in distinct IMP superfamilies using smFRET methods. Our experience includes studies of the neurotransmitter sodium symporter (NSS) family members LeuT and MhsT (51, 180, 211, 212); the excitatory amino acid transporter (EAAT) family member GltPh (3, 4, 31); HIV1 envelope proteins (ENV) (119, 120, 136); the P-type ATPase transporter family member LMCA1 (46, 48); the GPCR β_2_-adrenergic receptor (β2AR) (11, 12, 61); and the cystic fibrosis transmembrane receptor (CFTR), a member of the ABC transporter family (110) (Figure 1). Rather than recapping the detailed findings of these specific bodies of work, we broadly summarize the practical and conceptual advances that have been achieved and that are likely to be helpful to others. In this context, we share our perspectives on how to best ensure success in smFRET imaging endeavors and the general principles of metastability that we have discerned from our investigations, as well as frontiers for future progress in the field.

We refer the reader to the first reports of smFRET investigations of IMPs, which studied the *Escherichia coli* F_O_F_1_ ATP synthase (22) and Lactose permease (LacY) (123, 172); recent smFRET studies of the KirBac1.1 potassium channel (192); and related reviews in the field (20, 23, 42, 98, 127, 205). IMP functional insights have also been advanced by photoisomerization-induced fluorescence enhancement (PIFE) studies (77, 102, 150, 193) and pH-based single-molecule activity assays (97, 185, 189); for brevity, these topics are not covered in this review.

## STATIC STRUCTURES MOTIVATED THE EXPLORATION OF DYNAMIC PROPERTIES OF INTEGRAL MEMBRANE PROTEINS

Polytopic IMP structures, the essential foundation for smFRET interrogations of IMP activities, came into focus through pioneering biochemistry, neutron scattering, X-ray crystallography, and cryo-electron microscopy (cryo-EM) methods focused on native systems of relatively high abundance (186). These systems, which were particularly stable in detergent micelles, included IMPs mediating energy conversion (bacteriorhodopsin), vision (rhodopsin), membrane potential (ion channels), calcium transport [Sarco/Endoplasmic Reticulum CAlcium (SERCA)-type and other P-type ATPases], and cellular respiration (F- and V-type ATPases), as well as photosynthetic reaction centers (PSI and PSII). Advances in X-ray crystallography set in motion for soluble proteins by Perutz, Kendrew, and others ultimately led to the first structure of a bacterial photosynthetic reaction center in the mid-1980s (39, 40). Structures of lower-abundance IMPs, including light-activated GPCRs, bacteriorhodopsin, and bovine rhodopsin, were revealed near the end of the twentieth century, first by cryo-EM at modest resolution (62, 183) and then at atomic resolution by both traditional and lipidic cubic phase X-ray crystallography methods (59, 145, 148). Structures of the bacterial potassium ion channel KcsA (45, 214) and the yeast cytochrome *bc*_1_ complex (104) were solved contemporaneously.

As the field of molecular biology developed, researchers were enabled to heterologously express low-abundance, disease-associated IMPs, including the hormone-regulated GPCR β2AR (154). These efforts subsequently led to elucidation of how β2AR engages with intracellular heterotrimeric stimulatory G (G_s_) protein (155). The contemporaneous explosion of genomics information simultaneously enabled independent labs to determine structures of bacterial orthologs of critical mammalian IMPs, including LeuT and MhsT (NSS), Glt_Ph_ (EAAT), and LMCA1 (SERCA-type P-type ATPases) (47, 113, 124, 181, 202, 206). These first static IMP snapshots, together with a tsunami of high-resolution structures for myriad other systems (54), led to the realization that IMP functions likely require time-dependent changes in conformation. The capture of IMP structures in alternative conformations within a given family emboldened researchers to ask how they could experimentally gain kinetic and structural insights into the conformational changes underpinning IMP function. Questions of this nature gave rise to the field of smFRET imaging [originally referred to as single-pair FRET (spFRET)], which began in the late 1990s with the detection of individual, distinctly sized DNA oligonucleotides in aqueous buffers (66). Shortly after this seminal advance, efforts to extend smFRET imaging to diverse biological systems in increasingly physiological settings began in earnest (83).

## ESSENTIAL FOUNDATIONS FOR SINGLE-MOLECULE IMAGING INVESTIGATIONS OF INTEGRAL MEMBRANE PROTEINS

Static structural information is essential to guiding the placement of suitable donor and acceptor fluorophores at positions likely to generate information about compositional or conformational processes of interest in a given system (106, 107, 177, 194). In our own initiatives, we give preferential consideration to peripheral positions of low conservation and solvent accessibility. Prioritizing labeling sites based on physical intuitions and binding partner considerations has been found to be vital to measuring physiologically relevant processes. While only one structure of a given target of interest may be available at an investigation’s onset, it is increasingly common to identify informative sites of labeling using available or predicted (using tools like AlphaFold and RosettaFold) (18, 167) three-dimensional structures of the same or similar molecules with alternative conformations (170). Informative fluorescence resonance energy transfer (FRET)-labeling sites are defined in this review as those that are capable of revealing time-dependent structural changes specifically related to functional reaction coordinates.

The informative qualification requires that the donor- and acceptor-labeled (i.e., FRET-labeled) systems retain full functionality. Potential FRET-labeling sites are typically screened for rapid fluorophore tumbling and desirable photophysical behaviors. FRET-labeled constructs are then examined at the single-molecule scale to correlate one’s observations with bulk properties or behaviors. FRET-labeled systems that respond appropriately to ligand binding or mutations known to affect function are funneled into more in-depth interrogations, where the full dynamic range of biological variables can be assessed. Although time consuming and laborious, these initial screening steps are critical to ensuring that only viable FRET-labeled sensors of specific biological function are pursued.

One obvious and profound advantage of single-molecule investigations is the relatively scant demand for samples (approximately picomole to nanomole amounts) compared to other biophysical methods. This consideration has been particularly advantageous for investigations of IMPs derived from mammalian systems. In our experience, informative FRET-labeled samples can be prepared at modest scale, stored in frozen aliquots, and continuously interrogated for years.

## SITE-SPECIFIC LABELING OF INTEGRAL MEMBRANE PROTEINS

Small-molecule organic fluorophores serve as the most reliable extrinsic contrast agents for site-specifically labeling IMPs for single-molecule fluorescence and smFRET studies. This is due to their relatively small size (comparable to a dinucleotide or small peptide) and their efficient, regular, and durable photon emission (86, 106, 126, 213). Site-specific labeling can be performed in situ (81), within living cells (63, 179), in partially fractionated membranes (128), or on purified samples (e.g., within detergent micelles, reconstituted nanodiscs, bicelles, and proteoliposomes). In our smFRET investigations of IMPs, we have sought to demonstrate correlations with the three-dimensional structures and previous functional studies performed in detergent micelles and/or proteoliposomes.

A multitude of donor and acceptor fluorophores have been validated as being robust for smFRET imaging applications (2, 70). However, particular organic fluorophore species can exhibit physical or photophysical advantages and disadvantages for specific applications (67, 146). In our experience, planar hydrophobic organic fluorophores, such as ATTO647 and related species, while remarkably photostable in oxygenated environments, have the potential for nonspecific binding and spurious photon emission in deoxygenated and redox-active environments (146). When investigating IMPs, preference should also be given to fluorophores that exhibit reduced occupancies in long-lived, nonfluorescent triplet states, and that do not react with molecular oxygen to generate toxic reactive oxygen species (126, 213), to ensure that signals reporting on biophysical processes dominate over photophysical artifacts (67). Fluorophores of this kind are also beneficial for investigations of IMPs in membrane environments, as the solution additives typically utilized to exogenously suppress triplet states are membrane perturbing (5) and highly toxic to cells (146). Self-healing cyanine-class organic fluorophores leveraging intramolecular Baird aromaticity-promoted, triplet–triplet energy transfer (TET)-based quenching mechanisms (8, 9) have proven to be robust options for smFRET investigations of IMPs, as they are also particularly suited to interrogations of individual molecules at rapid timescales and over extended periods (5, 146). Reviews of self-healing organic fluorophores can be found elsewhere (126, 213).

**Figure 2:**
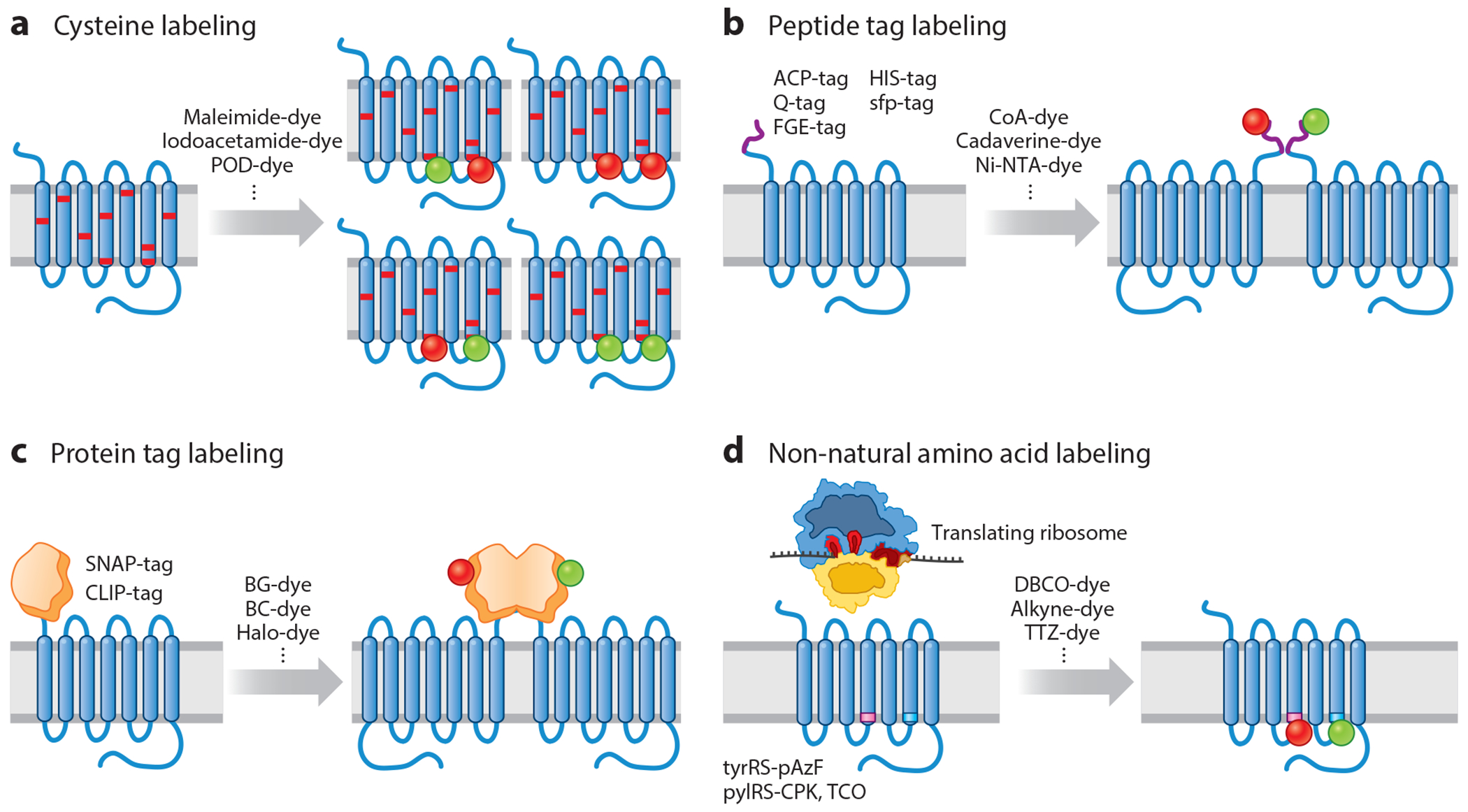
Site-specific labeling of integral membrane proteins (IMPs) for single-molecule fluorescence resonance energy transfer (smFRET). IMPs, as represented by a schematic of a G protein–coupled receptor (GPCR), can be site-specifically labeled with fluorophores via (*a*) engineered cysteines, (*b*) peptide tags, (*c*) protein tags, and (*d*) non-natural amino acids.

Site-specific labeling of IMPs with organic fluorophores can be achieved via a multitude of long-held or emerging chemical strategies. Intrinsic or site-specifically introduced cysteine residues can be stochastically labeled with both donor and acceptor fluorophores using thiolreactive chemistries (93, 210) (Figure 2a). However, this approach can suffer from substoichiometric labeling and/or nonspecific labeling of native cysteines in the target protein. The removal of native cysteines and/or the introduction of cysteines at solvent-accessible sites in a target protein requires careful assessment that function is maintained. Although stochastic cysteine labeling reduces the proportion of the FRET-labeled population (proteins bearing only a single donor and a single acceptor fluorophore), they can be computationally separated after image acquisition (4, 46, 48, 180). When native cysteines must be present to preserve function, iodoacetamide chemistries can potentiate selective labeling of the most solvent-accessible, and thus reactive, cysteine residues (61). As cysteine residues are often prevalent and functionally important in mammalian IMPs, likely due to their roles in protein lipidation (60), structural integrity (89), redox sensitivity (19), and protein–protein interactions (130), alternative labeling strategies will likely be required to advance investigations in this area.

Genetic fusion of engineered peptide tags into target proteins has recently emerged as a powerful alternative labeling strategy. Such methods require the introduction of short (approximately 4–12 amino acid) peptides, such as the ACP-tag (10, 55), Q-tag (112), FGE (Formylglycine Generating Enzyme)-tag (27, 169), or HIS-tag (87), into the protein of interest, which can then be linked to activated organic fluorophores such as coenzyme A-, cadaverine-, aldehyde-, or Ni-NTA-activated fluorophores via either self-activated or enzyme-mediated conjugation (Figure 2b). FRET-labeling of complex systems has been achieved by employing peptide tags in combination at the N or C terminus (191) or within internal loops or unstructured regions (136). Analogous outcomes can be achieved using self-activating protein domains such as SNAP, CLIP, and Halo (using BG-, BC-, and chloroalkyl-activated fluorophores, respectively) (176), although these protein tags (approximately 20 kDa) are significantly larger in size than short peptides. Due to their larger size, the attachment of SNAP-, CLIP-, and Halo-tags is generally restricted to the termini of the proteins (11, 184) (Figure 2c). Constructs created using either method require functional screens.

The advent of genetic code expansion in the early twenty-first century enabled site-specific incorporation of non-natural amino acids (nnAAs) with bioorthogonal labeling chemistries, ostensibly at any position within a target protein (103). Progress on this front was advanced by the discovery of a reprogrammed genetic code in methanogenic Archaea (139), which are orthogonal to both bacterial and eukaryotic host cells (Figure 2d). The high precision of nnAA incorporation into otherwise native systems, together with the small size of nnAAs, makes genetic code expansion methods ideal for single-molecule fluorescence and smFRET investigations. Successfully incorporated nnAAs can be subsequently conjugated through Schiff-base chemistry using hydrazide- or hydroxylamine-activated fluorophores (35, 50), inverse electron-demand Diels-Alder reactions with tetrazine- or methyltetrazine-activated fluorophores (21, 100, 121, 208), strain-promoted azide-alkyne click chemistry reactions using DBCO- or BCN-activated fluorophores (44, 65), or photocrosslinking (33, 166).

Although nnAA incorporation has been reported for smFRET investigations of specific IMPs (160), current nnAA technologies generate relatively poor yields of full-length protein (38). The site-specific labeling yields of these chemistries are also often substoichiometric. Overcoming these practical limitations will likely require advances in orthogonal translation machinery expression (28, 29, 82, 95, 96, 114, 168, 190, 203) and cell-strain engineering (101, 116, 131, 142, 156, 164, 168, 203), as well as the optimization of biorthogonal chemistries for quantitative labeling yields (165). nnAA technologies leveraging the principles of genetic code expansion in vitro (36) or the injection of orthogonal translation components into living cells (24, 79, 116) also hold promise. Ultimately, advances on these fronts must aim to generate a single polytopic IMP containing two distinct nnAAs to enable site-specific labeling with both donor and acceptor fluorophores using orthogonal conjugation chemistries, combined with subsequent purification strategies that will yield only the quantitatively FRET-labeled samples.

## PLATFORMS FOR SINGLE-MOLECULE IMAGING OF INTEGRAL MEMBRANE PROTEINS AND OTHER BIOLOGICAL SYSTEMS

**Figure 3:**
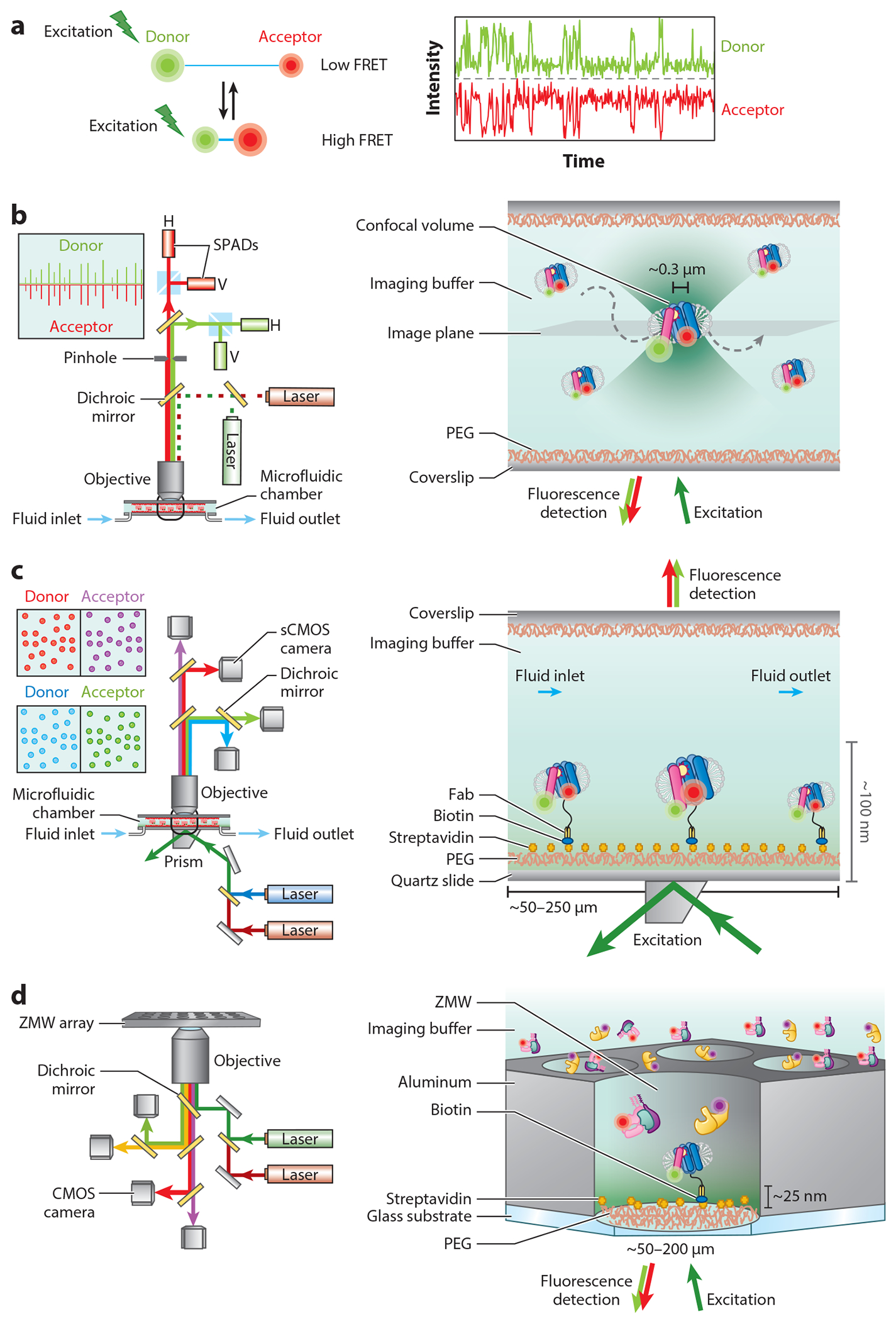
Single-molecule imaging platforms. (*a*) (*Left*) Distance-dependent FRET interaction (*blue bar*) between a donor (*green*) and an acceptor fluorophore (*red*). (*Right*) Fluorescence intensity traces of donor and acceptor fluorophores. (*b–d*) Schematic representation of (*b*) confocal, (*c*) TIRF-, and (*d*) ZMW-based smFRET imaging platforms. Abbreviations: CMOS, complementary metal-oxide-semiconductor; FRET, fluorescence resonance energy transfer; sCMOS, scientific CMOS; smFRET, single-molecule FRET; SPAD, single-photon avalanche diode; TIRF, total internal reflection fluorescence; ZMW, zero-mode waveguide. Figure created in part using images from Biorender.com.

The imaging of single fluorescent- or FRET-labeled biomolecules was first made possible by confocal and total internal reflection (TIR) fluorescence microscopy (TIRFM) platforms, which were later complemented by zero-mode waveguide (ZMW) technologies (Figure 3). All three techniques dramatically improve (ca. 20–50-fold) the signal-to-noise ratio of image detection by reducing out-of-focus (background) fluorescence and allow the tracking of donor and acceptor fluorophore positions in space and time, as well as the distance between them, via FRET (Figure 3a).

Confocal microscopes (Figure 3b), first developed in the mid-1950s (132), focus incident light to a narrow waist and selectively remove out-of-focus light via the careful placement of a pinhole in a conjugate plane. Confocal systems are typically used to monitor individual molecules during their diffusion through a sub-femtoliter-sized imaging volume, which can be slowed using anti- Brownian electrokinetic (ABEL) traps (32). In the mid-1970s, confocal microscopy was brought to bear by Watt Webb’s team to image fluctuations in individual biomolecules (122), and it continues to be widely implemented for an increasingly broad array of investigative pursuits (43). In smFRET investigations, confocal illumination strategies can be implemented with continuous excitation, alternating laser excitation (ALEX), or pulse-interleaved laser excitation (88, 133) to excite diffusing or surface-immobilized molecules. Fluorescence emission is typically detected using single-photon avalanche diodes (SPADs), sensitive photoelectric chips capable of detecting the timing of single photon arrivals with picosecond resolution. Such methods are capable of leveraging single photon counting statistics and fluorescence lifetime and/or polarization measurements simultaneously. Multiparameter smFRET measurements that provide simultaneous FRET and fluorescence lifetime or polarization measurements have become increasingly popular for investigations of conformational processes occurring on the microsecond to millisecond timescales (117, 201).

In the early 1980s, Daniel Axelrod, who also worked with Webb, developed TIRFM to examine biological processes occurring at or near the cell membranes (14–16) (Figure 3c). TIRFM projects a relatively broad laser beam greater than the critical angle to a glass-aqueous solution interface to generate a relatively large-area (up to 375 × 375 μm^2^), exponentially decaying evanescent field that can be used to excite fluorophores proximally localized within approximately 100–200 nm from the point of reflection. TIRFM has since become a powerful tool to study single-molecule phenomena, including the movements of individual molecules on the surface of living cells, protein dimerization, and membrane trafficking (11, 90, 188). TIR can be generated via multiple strategies, with objective-based and prism-based methods being the most widely employed. Both methods require confinement of the FRET-labeled species of interest within the evanescent wave generated by TIR. For cellular studies, this typically limits observations to the portion of the cell surface that is adherent to robustly cleaned and appropriately activated quartz substrate. For in vitro investigations, TIRFM stipulates implementation of biochemical strategies to stably tether FRET-labeled samples near a cleaned and passivated quartz surface. In objective-based TIRFM, excitation and emission pathways are transmitted through the magnifying objective lens. In prism-based TIRFM, incident excitation occurs opposite to the objective lens (Figure 3c). Fluorescence emission in both configurations is typically projected onto electron-multiplying charge-coupled device cameras. Scientific complementary metal-oxide-semiconductor (sCMOS) cameras have also been recently implemented, which offer larger imaging fields, faster readout rates, and improved signal-to-noise ratios compared to EMCCD cameras (85). Using sCMOS technologies, experimental throughput can be increased by up to an order of magnitude to simultaneously observe tens of thousands of individual molecules (85).

In the early 2000s, Webb’s team implemented ZMWs to enable observation of DNA polymerase–mediated synthesis of double-stranded DNA, which require the use of high (micromolar to millimolar) concentrations of fluorescently labeled nucleotides, multiple orders of magnitude greater than those permitted in confocal or TIRFM setups (108). ZMWs are nanoscopic wells approximately 50–200 nm in diameter that create evanescent fields at glass-aqueous solution within tiny volumes in which only a handful of single molecules are present at high concentrations (Figure 3d). ZMW technologies have since been successfully advanced for real-time (e.g., single-molecule real-time sequencing) techniques for DNA sequencing (49), protein synthesis reactions (182), and ligand-binding studies (195). While ZMWs can be advantageous in specific settings and applications, the required metal-coated glass is difficult to obtain and has the potential to create exaggerated nonspecific binding challenges, as well as background fluorescence. However, a potential advantage of this technique is that it can generate large, ordered arrays that contain thousands to hundreds of thousands of individual ZMWs, which in principle enables highly parallelized measurements. Moreover, plasmon-induced fluorescence enhancements that can sometimes arise through the use of ZMWs have the potential to significantly increase the brightness of organic fluorophores (53). However, the full advantage of large array sizes is limited by Poisson statistics, which stipulate that only 37% of ZMWs contain only a single molecule (149). Fluorescence enhancements can also be highly variable, adding to experimental noise (196).

Prism-based TIRFM platforms equipped with sCMOS cameras have emerged as the primary workhorses in our efforts to investigate the dynamic properties of individual IMPs. Such systems offer experimental throughputs that are multiple orders of magnitude greater than confocal systems and somewhat higher than ZMW-based systems (approximately 2–10-fold). Prism-based TIRFM platforms are also particularly suited to buffer exchange, which is critical to examining a system’s responses to stimuli or when the order and timing of events in nonequilibrium processes must be established (85). Confocal measurements are selectively employed to establish target molecule diffusion rates, fluorescence lifetimes, or photophysical information and to define dynamic processes faster than those accessible via TIRFM imaging.

## PRACTICAL CHALLENGES IN RELATING FLUORESCENCE RESONANCE ENERGY TRANSFER EFFICIENCY MEASUREMENTS TO DISTANCE

Regardless of the experimental approach, the goal of imaging a FRET-labeled target is to convert time-dependent changes in donor and acceptor emission intensities for an individual molecule to distances that reflect time-dependent compositional and conformational changes (Figure 3a). Standardized procedures have been established to aid in the conversion of donor and acceptor fluorescence intensities into the desired time-dependent changes in FRET efficiency and distance information (106). Quantitative analysis tools are then applied to these data to delineate the repertoire of transient states evidenced, the order and timing in which they are transited, and the rates of transitions between them.

While a variety of analytical approaches and tools are available to access such information that may each have their own advantages and disadvantages in distinct settings (58), we have had consistent success across a variety of biological systems with hidden Markov modeling algorithms originally developed for ion channel recordings (135, 151). These algorithms, along with tools for data processing and visualization, have been reconstituted within the SPARTAN software platform to provide users with a complete analysis pipeline (85).

FRET measurements generally require several hundred total photons per given time interval to achieve signal-to-noise ratios sufficient to robustly determine a FRET efficiency value. Given the approximately 10% photon detection efficiency for modern imaging systems, this equates to a fluorophore photon emission rate of approximately 10,000,000 s^−1^ (10,000 photons ms^−1^) at the practical resolution limits of smFRET investigations (approximately 100–200 μs) (146). For cyanine fluorophores that have nanosecond fluorescence lifetimes, this equates to just 2–10% of their maximum photon emission rate. To reach such rates, robust suppression of fluorophore photophysical processes and photobleaching is paramount (85, 146).

To avoid dramatic reductions in the size of the imaging field, and rapid photobleaching, we typically initiate sCMOS-based TIRFM investigations at an imaging rate of 100 frames s^−1^ (10 ms time resolution). At this frame rate, under optimized conditions, the biological functions of individual molecules can be imaged over extended periods (approximately tens of seconds), where distance changes on the sub-nanometer scale (approximately 5 Å) can be reliably measured when donor and acceptor fluorophores are separated by approximately 20–80 Å (2, 70, 158). When possible, we employ self-healing cyanine-class fluorophores implementing TET technologies to enhance the precision, accuracy, and resolution limits of smFRET (147).

## STRUCTURE–FUNCTION RELATIONSHIPS AT THE SINGLE-MOLECULE SCALE

Historically, scientists have investigated molecular function by measuring the rates at which products are generated and downstream reactions are triggered. For instance, radioactive ligand-uptake assays played a vital role in examining the activity of transporter and/or channel proteins at the ensemble scale. The goal of directly imaging biological function from the perspective of time-dependent changes in fluorescence and FRET in individual molecules inverts the focus onto the enzyme itself. In this reference frame, single-molecule activities distill to time-series information that reveals the order and timing of conformational and compositional events and uncovers the relationship between these events and functional outcomes.

**Figure 4:**
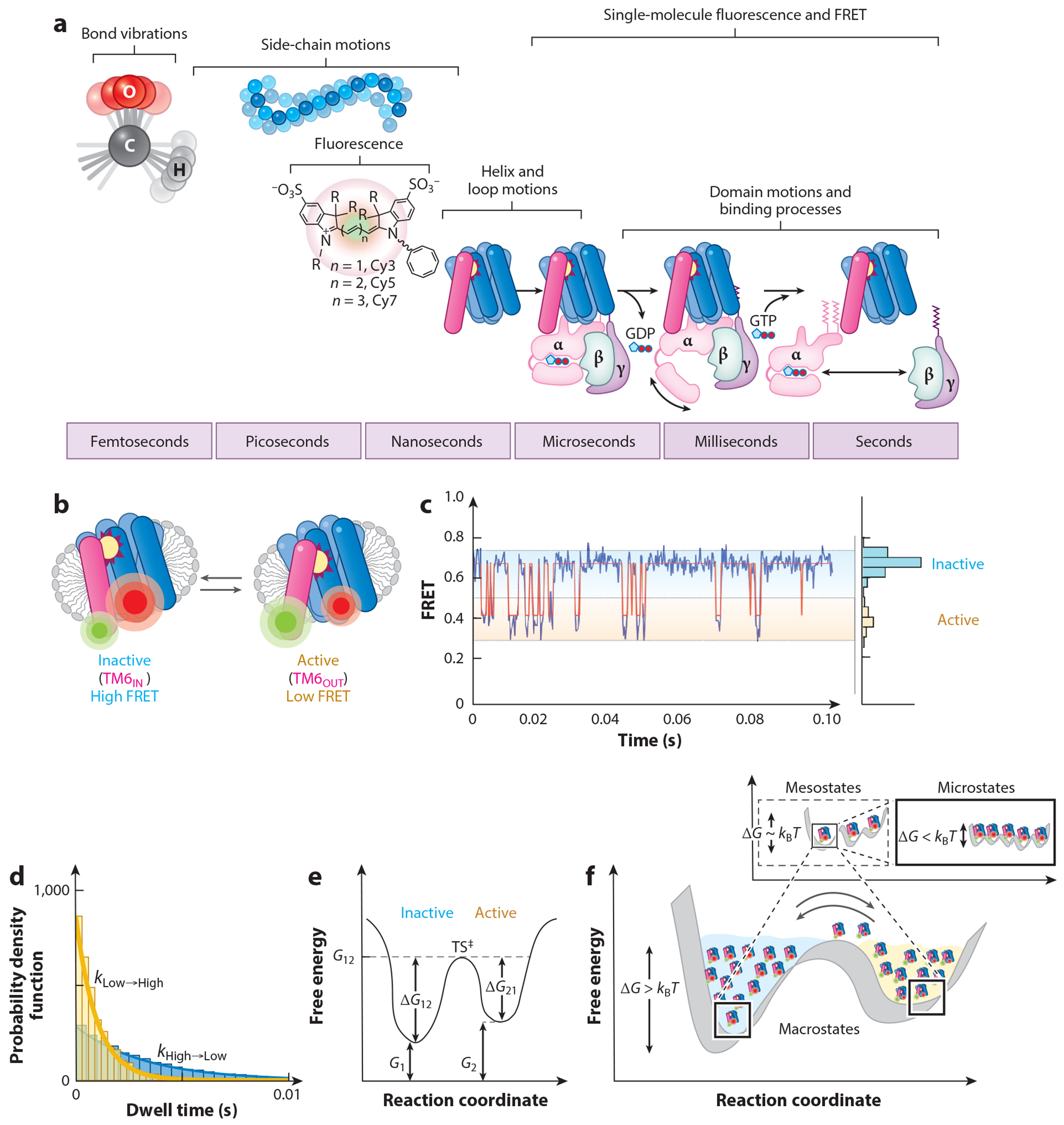
Quantifying integral membrane protein (IMP) metastability at steady state. (*a*) Timescales of protein motions exemplified with G protein–coupled receptor (GPCR)-mediated signaling events (on a nanosecond to second scale) that can be monitored by single-molecule fluorescence resonance energy transfer (FRET). (*b*) Schematic of single-molecule FRET imaging of TM6 motions of agonist-bound β_2_-adrenergic receptor (β2AR). (*c*) Simulated single-molecule FRET signal (*blue trace*) with idealization (*red line*) of agonist-induced TM6 motions at an integration time of 250 μs. (*d,e*) Estimation of the relative free energies of inactive (state 1) and active (state 2) β2AR from experimental dwell time information (*d*), interpreted as relative free energy parameters, including the transition state barrier (TS^‡^) height, where $k_{\text{High}\to \text{Low}}=C_{1}e^{-\frac{\Delta G_{21}}{k_{B}T}},k_{\text{Low}\to \text{High}}=C_{2}e^{-\frac{\Delta G_{12}}{k_{B}T}}$ (*e*). (*f*) In addition to this information, high-temporal- and -spatial-resolution single-molecule FRET data, and data collected over extended periods, can reveal additional, functionally relevant features of the energy landscape, including mesoscopic $\left(\Delta G∼k_{B}T\right)$ and microscopic $\left(\Delta G<k_{B}T\right)$ roughness within each macroscopic $\left(\Delta G>k_{B}T\right)$ basin. Figure created in part using images from Biorender.com.

At ambient temperatures, biomolecules are in constant motion, ranging from femtosecond-timescale bond vibrations to millisecond- to second-timescale conformational and compositional processes (Figure 4a). As organic fluorophores absorb and emit photons in the picosecond to nanosecond regime, information in single-molecule fluorescence and smFRET is generally limited to events that occur on significantly longer timescales, such as helix and loop motions, domain motions, and binding events, in which intra- or intermolecular contacts are formed and/or broken. These relatively slow, thermally driven processes give rise to changes in the absolute and relative positions of the rapidly tumbling fluorophores at their respective labeling sites, which are ideally suited for camera-based smFRET measurements.

In the hyperthermophilic NSS and EAAT orthologs, LeuT and GltPh, respectively, we observed relatively stable, ground-state configurations under steady-state conditions, with FRET values recapitulating the outward-facing transporter conformations observed crystallographically (3, 4, 51, 57, 180, 211, 212). Optimized buffer conditions, including solution additives that quench fluorophore triplet states, enabled detection of spontaneous and transient conformational processes on the minute timescale. In the presence of transported ligands, these spontaneous processes were potentiated; in the presence of inhibitors, they were blocked. Targeted, structure-informed mutational analyses, combined with structural and molecular dynamics assessments, led to assignment of these dynamics to gating (LeuT) and elevator-like transport domain (GltPh) processes (3, 57, 211). When contacts critical to a specific conformation were perturbed, occupancy of the corresponding FRET state was reduced. The opposite held true when stabilizing contacts within a specific conformation were formed. Assigning experimentally observed FRET values to specific conformational states of a system is vital to relating the occupancies of each conformational state to function.

## QUANTIFYING FUNCTIONAL DYNAMICS AND METASTABLITY IN INTEGRAL MEMBRANE PROTEINS

To illustrate this point, we use the activation of β2AR, a prototypical class A GPCR, as an example (Figure 4b). GPCRs, constituting the largest human superfamily of cell surface receptors (163), are critical for triggering cellular responses to extracellular signals by transiently interacting with diverse intracellular proteins, including heterotrimeric G proteins (G_s_, G_q_, G_i/o_, G_12/13_, etc.), arrestins, and kinases (73). Each of these interactions entails coordinated conformational events in both the GPCR and its binding partners. In the case of heterotrimeric G proteins, these events are vital to both nucleotide exchange and heterotrimer dissociation processes (61). Extant literature has led our team and others to hypothesize that ligand-specific differences in the rates and extents of activating conformational changes in the receptor and/or receptor binding partner complexes are critical to signaling fidelity and signaling strength.

In our initial smFRET investigations of β2AR, we site-specifically labeled detergent-solubilized protein at the intracellular termini of transmembrane helix 4 and transmembrane helix 6 (TM6) (see 61). Based on prior biophysical investigations, we sought to image and quantify transient outward deflections of TM6 induced by agonist binding to the orthosteric site within the extracellular vestibule to correlate these motions with heterotrimeric G protein coupling. To make these investigations possible, we utilized a custom-synthesized donor Cy3B fluorophore, in which rigidification and charge distribution had increased the flux and regularity of the photon yield, combined with a self-healing Cy7 fluorophore derivative exhibiting substantially greater brightness and total photon yield than the parent Cy7 fluorophore. While these fluorophores were optimally tuned for β2AR’s relatively small size and the scale of the conformational change to be detected, they limited our imaging time resolution to a frame rate of 100 s^−1^ (at a 10-ms exposure period). At this time resolution, the predominant conformation observed was consistent with crystallographic structures of β2AR in the inhibited state (high-FRET efficiency). When partial and full agonists were added, the observed FRET value decreased in an agonist-dependent fashion, consistent with the expected movement of the TM6 of the receptor away from the central helical bundle (Figure 4b). Clearly distinguishable dynamic processes were not, however, readily evidenced. Instead, agonist potency correlated with changes in the amplitude of TM6 movements. For the synthetic partial agonist, salbutamol, and the cognate agonist, adrenaline, we estimated that the outward deflection of TM6 was on the order of approximately 2–4 Å on a time-averaged basis. Importantly, the strength of these TM6 movements correlated with heterotrimeric G_s_ protein coupling efficiency.

In contrast, findings from X-ray structures, electron spin resonance studies, and comparative molecular dynamics simulations of fluorophore-labeled β2AR protein (61, 125, 155) suggested a much larger, approximately 14-Å outward shift in TM6 position in the activated state (e.g., low-FRET efficiency). These contrasting findings led us to perform fluorescence cross-correlation analyses to explain the experimental data. We proposed that larger-amplitude movements of TM6 occurring at rates much faster than our time resolution (≥300 s^−1^) could account for the observed data. Kinetic simulations reveal that collecting smFRET data at a frame rate of 4,000 s^−1^ (0.25 ms integration time) would therefore be necessary to robustly capture TM6 movements to fully activated positions (Figure 4c). In this time regime, the lifetimes of transient TM6 deflections and the transition rates between them (*k*_High→Low_ and *k*_Low→High_) can be determined (Figure 4d). From these data, the relative free energies of inactive (Δ*G*_12_) and active (Δ*G*_21_) β2AR conformations can be estimated based on the fundamental relationships between rates and free energies defined by the Arrhenius equation (Figure 4e):

1. $$k_{\text{High}\to \text{Low}}=C_{1}e^{-\frac{\Delta G_{21}}{k_{B}T}},\;k_{\text{Low}\to \text{High}}=C_{2}e^{-\frac{\Delta G_{12}}{k_{B}T}},$$

where *C*_1_, *C*_2_ are prefactors associated with barrier-crossing attempt frequencies, and *k_B_* and *T* are the Boltzmann constant and temperature, respectively (197).

It is our goal to perform TIRFM imaging of β2AR and other GPCRs at sufficient time resolutions to precisely resolve this information and to differentiate between the distinct models of TM6 motion. To date, however, we have only been able to achieve 4,000 s^−1^ (a time resolution of 250 μs)–10,000 s^−1^ (a time resolution of 100 μs) imaging rates for model systems in our lab (146). Access to this time domain, given sufficiently high signal-to-noise ratios, would enable us to quantitatively assess the kinetic, energetic, and structural impacts of distinct agonists and antagonists. This information should generate new insights into the mechanisms of activation and downstream signaling, as well as generating new hypotheses about the nature and complexity of micro-, meso-, and macroscopic energy basins within the metastable landscape and the barriers among them (72, 129, 197, 198) (Figure 4f). Further structural and mechanistic insights into inactive and active conformations, as well as the TS^‡^ between them, could be obtained through a combination of mutational and linear free energy relationship [also known as Phi (Φ)] analyses (1, 26, 76, 80, 138). While studies of this kind, in combination with molecular dynamics simulations, have proven highly informative in our investigations of LeuT and GltPh (3, 4, 57, 180, 211, 212), it remains a possibility that TM6 does not exhibit rapid transitions between inactive and active states, but instead exhibits discrete, outward-deflected positions that vary in extent depending on the bound agonist. Future studies are required to resolve these distinct models of β2AR activation and the mechanism enabling specific heterotrimeric G protein coupling, guanosine nucleotide exchange, and heterotrimer dissociation processes.

## THE PREDICTIVE POWER OF SINGLE-MOLECULE FLUORESCENCE RESONANCE ENERGY TRANSFER TO CAPTURE STRUCTURAL DYNAMICS IN INTEGRAL MEMBRANE PROTEINS

Steady-state smFRET investigations of LeuT; GltPh; and, most recently, the human ABC transporter family member CFTR have validated the smFRET approach as a powerful tool to identify transient states critical to function that would be difficult or impossible to discern through ensemble methods. With this information, in combination with theoretical approaches and molecular dynamics simulations (25, 56, 110, 197, 198, 209), IMP structure–function relationships have been examined and revealed.

For instance, smFRET investigations revealing alternative conformations of LeuT and GltPh have been used to guide and interpret static structure determination efforts and outcomes. For LeuT, our initial observations of TM1a movements by smFRET, combined with mutational analyses and molecular dynamics simulations, predicted that its outward movement is responsible for intracellular gate opening, yielding a key inward-facing intermediate (211). Structural capture of inward-facing LeuT conformations later confirmed this prediction (57, 64, 91, 92, 99, 174). Similar conclusions have been drawn for other NSS family proteins (84). In the case of GltPh, smFRET data documented elevator-like movements of the independent transport domains, which had been hypothesized based on mercury cross-linked structures (3, 4, 134, 157). Analogous structure determination efforts for the P-type ATPase LMCA1 (46, 48) and CFTR (110, 207) have also captured intermediate states first identified through smFRET investigations. Parallels between smFRET and static structures have also been demonstrated for distinct biochemical systems (78, 141, 153, 200), including ribosomes (30, 34, 74, 159). In our collaborative investigations of the HIV ENV, smFRET studies have predicted the existence of a ground state (state 1) that is specifically stabilized in the context of the intact virion, which has yet to be captured by static structural methods (111, 118). Continued efforts to establish concrete connections between smFRET and static three-dimensional structures constitute a vital frontier for establishing smFRET imaging as a robust biophysical and structural tool in the researcher’s arsenal.

## IMPLICATIONS OF INTEGRAL MEMBRANE PROTEIN METASTABILITY

**Figure 5:**
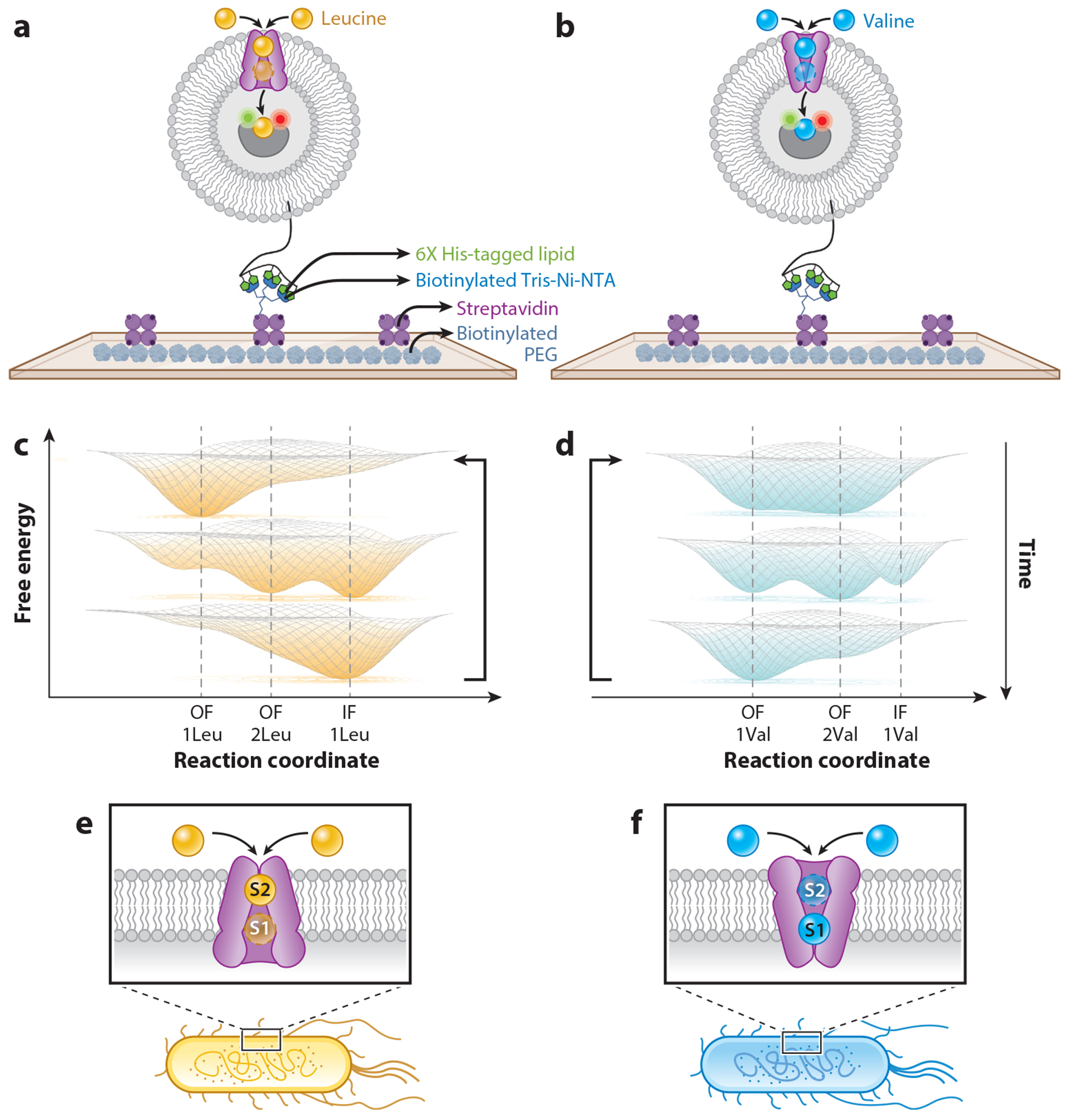
Bridging integral membrane protein (IMP) metastability with function. (*a*,*b*) Experimental design of a single-molecule fluorescence resonance energy transfer (smFRET)-based pre-steady-state assay to examine uptake of the amino acid [(*a*) leucine or (*b*) valine] by MhsT. (*c*–*f*) Schematic representations of the free energy landscapes for MhsT transporting (*c*) leucine and (*d*) valine. The substrate (leucine or valine) binds to both the orthosteric (S1) and allosteric (S2) binding sites and modifies MhsT transitions between (*e*) inward-facing (IF) and (*f*) outward-facing (OF) states. This prediction was supported by a live-cell chemical probing assay. Figure created in part using images from Biorender.com.

In addition to the aforementioned steady-state analyses, valuable mechanistic information can also be revealed using pre-steady-state smFRET investigations that assess single- or multiturnover reactions. In such studies, the energy landscape can sequentially change in response to ligand-induced conformational processes and functional outcomes. To illustrate this point, we draw the reader’s attention to our recent efforts using single-molecule TIRFM to quantify the activities of a bacterial homolog of the eukaryotic NSS proteins, MhsT, a transporter of hydrophobic amino acids (e.g., Ile, Val, and Leu) (see 51). In this investigation, we encapsulated a FRET-labeled hydrophobic amino acid binding protein, the leucine, isoleucine, valine periplasmic binding protein (LIV-BP), within the lumen of proteoliposomes to measure the MhsT transport activity (Figure 5). After being transported into the proteoliposome lumen, the amino acid binds to a single luminal LIV-BP, causing it to adopt a compact conformation (Figure 5a,b) and triggering either an instantaneous or a gradual concentration-dependent increase in FRET, depending on whether LIV-BP exhibits high affinity (wild type) or low affinity (mutant) for the hydrophobic amino acid, respectively. Correspondingly, wild-type LIV-BP enabled single-turnover pre-steady-state kinetic measurements; mutant LIV-BP enabled multiturnover, pre-steady-state kinetic measurements. Using these assays in combination, we unexpectedly revealed differences in the rate-limiting features of the MhsT-mediated transport of leucine and valine amino acids. For leucine, the rate-limiting step was isomerization of the protein from an inward-facing conformation, subsequent to amino acid release, to an outward-facing conformation capable of additional transport cycles. For valine, the rate-limiting step was isomerization of the outward-facing protein to an inward-facing conformation capable of substrate release into the lumen. From these kinetic data, and prior experiments on the evolutionarily related LeuT transporter (51, 57, 180, 211, 212), we inferred that the dynamic energy landscapes traversed by the same protein when transporting leucine or valine amino acids were mechanistically distinguishable. This allowed us to predict that MhsT molecules actively transporting leucine would on average reside in an inward-facing conformation, whereas MhsT molecules actively transporting valine would on average reside in an outward-facing conformation (Figure 5c,d). These mechanistic inferences and predictions were supported by live-cell chemical probing assays tracking the inhibition sensitivity of cysteine groups within the intracellular vestibule of MhsT to membrane permeable N-ethylmaleimide.

A key question that these observations raise is how the rate-limiting step of the MhsT transport mechanism can depend on the nature of the amino acid that it transported. In other words, how can the rate-limiting transport step occur after the substrate is released to the lumen? We concluded from these investigations that MhsT, and perhaps other NSS family proteins (51), can bind two substrates, and substrate release of the more centrally located orthosteric binding site (S1) is stimulated by the binding of the second substrate to a proximally located allosteric binding site (S2) (Figure 5e,f). While this is an attractive model in light of extant literature (152, 171, 211, 215), the role of the allosteric substrate binding site in NSS family proteins remains controversial. The relationship between allosteric sites localized in a similar region of the extracellular vestibule human serotonin transporter (204) and the transport mechanism has yet to be examined. Direct smFRET evidence also has yet to capture the differences predicted by our studies in the time-averaged conformations of MhsT when transporting leucine versus when transporting valine.

Each of the smFRET investigations touched upon in this section highlights how evolved metastabilities can functionally manifest in IMPs and how functional assays at the single-molecule scale can reveal unexpected and previously masked mechanistic insights. In the case of our single-molecule transport assays, the utility of performing investigations on fully reconstituted systems, where conditions can be exquisitely controlled, can be profoundly important. In the case of MhsT, we would have been unable to discern unambiguously that the rate-limiting transport step varied for distinct substrates were we not able to add only one substrate type at a time in pure form. In the case of GltPh, we would have been unable to understand or examine the relationship between kinetic and transport rate heterogeneities (31).

Our recent collaborative investigations of fully phosphorylated β2AR provide another example of the power of precisely controlled settings. In this study, we were able to deconvolute the physiologically linked processes of agonist-induced phosphorylation by cellular kinases from the process of agonist-induced β2AR coupling with its cognate stimulatory heterotrimeric G protein (G_s_) and β-arrestin (12). Consequently, we were able to predict that the C-terminal phosphorylated tail element of β2AR remained sequestered and unavailable for β-arrestin coupling in the absence of receptor agonism. Auto-inhibitory contributions of the β2AR C-terminal tail were subsequently supported by targeted cellular investigations (12, 71). In both systems, the integrative nature of our investigations enabled us to extrapolate our in vitro results to the physiological context and thereby strengthen the conclusions that could be drawn.

## KEY FRONTIERS IN SINGLE-MOLECULE FLUORESCENCE RESONANCE ENERGY TRANSFER IMAGING

The implementation of smFRET, advanced by our collaborative team and others, to investigate functional dynamic processes has led to new mechanistic insights and new testable hypotheses across a range of distinct polytopic IMP family members. Access to the dimensions of structural dynamics and time has significantly aided efforts to delineate precise structure–function relationships in diverse IMPs and new appreciations of their exquisite sensitivities to subtle perturbations of their sequence (disease mutations) and environment (temperature, ionic strength, and lipids) and the binding of small molecules (cognate ligands and small-molecule drugs). Given that many more questions have arisen for the systems already explored, and that myriad IMPs critical to cellular physiology have yet to be examined, it is our perspective that we are in the early dawn of mechanistic investigations of IMPs at the single-molecule scale.

Our team’s preliminary studies of bacterial and mammalian IMPs highlight both the nascent potential of smFRET imaging for discovery and the current limitations of the field, areas in which progress will be needed for further advancement. For instance, our early studies were enabled by efforts to directly connect mechanistic smFRET investigations of highly purified components in controlled experimental settings to previously determined structures. Consequently, we focused much of our initial work on well-behaving IMPs that were amenable to solubilization, isolation, and manipulation in detergent systems. Parallel ensemble studies using reconstituted proteoliposomes proved critical, however, to linking dynamic processes to functional outcomes (3, 4, 211, 212). Technical advancements enabling access to the transport mechanisms of NSS and EAAT proteins using sensor-encapsulated proteoliposomes provided the next critical advance (31, 51). Efforts must now be made to perform direct measurements of NSS and EAAT conformational changes and transport processes at the same time through the implementation of multicolor FRET experiments. In such settings, functional dynamic processes within individual sensor and transport proteins in the context of a single liposome can be simultaneously measured and quantified.

Pursuits of this kind will be fundamentally enabled by advances in imaging platforms and organic fluorophores that facilitate multiwavelength, multiperspective investigations. Progress on these fronts will ensure that distinct molecular events can be simultaneously examined, such as the timing of agonist-induced TM6 movements in GPCRs and coupling with cognate G proteins and β-arrestins (Figure 4d) or conformational events underpinning the transport mechanism and the arrival of transported substrates by luminal sensors (Figure 5). Parallel efforts to facilitate or increase experimental and analytical throughput by way of platform engineering and automation procedures are likely to prove vital to this frontier. These efforts will enable more rapid searches for informative FRET-labeled sensors, as well as the screening of condition space to optimize activities and reaction rates of interest or to rapidly assess small-molecule interactions and the molecular determinants of function through mutagenesis. This information will be vital to expanding our fundamental knowledge of the functional consequences of environmental condition changes and disease mutations, while simultaneously providing potentially important clinical insights into how native and mutated IMPs may be physiologically and pharmacologically regulated with specificity.

Alongside work on these frontiers, it will remain vital to link outcomes and predictions drawn from mechanistic studies in vitro with cellular settings, where the physiological groundings of membrane, cytoskeletal, and regulatory networks are ensured. Controlled in vitro investigations of each variable are expected to add fundamental knowledge to our understanding of IMP metastability and ultimately close the gap between in vitro and cellular contexts, thus strengthening the field. While self-assembled lipid bilayer nanoscale structures, such as nanodiscs (41), represent a viable option for such pursuits and are more suitable to cryo-EM and other experimental techniques (13), reconstituted proteoliposome technologies offer the benefit of providing unconstrained environments while also enabling investigations of physiological electrochemical gradients, IMP–IMP interactions, and membrane leaflet–specific interactions or modifications. In a similar spirit, technologies advancing the means to rapidly and effectively isolate IMPs without the need for detergent solubilization so as to maintain their cellular contexts may also prove vital (17, 52, 178). Methods that directly capture site-specifically labeled IMPs from the cellular envelope for single-molecule interrogations may be particularly important in this regard (81, 109, 184, 187). Our collective goals must also include advances in imaging, site-specific labeling, and organic fluorophore technologies to enable robust interrogations of IMP dynamics and downstream signaling events within IMPs’ native cellular contexts (11, 161, 199).
